# Interpersonal justice climate, extra-role performance and work family balance: A multilevel mediation model of employee well-being

**DOI:** 10.1371/journal.pone.0207458

**Published:** 2018-11-20

**Authors:** Vicente Pecino, Miguel Ángel Mañas-Rodríguez, Pedro Antonio Díaz-Fúnez, José M. Aguilar-Parra, David Padilla-Góngora, Remedios López-Liria

**Affiliations:** 1 HRM Office & IPTORA Research Team, University of Almería, Almería, Spain; 2 Department of Psychology & IPTORA Research Team, University of Almería, Almería, Spain; 3 Department of Psychology, University of Almería, Almería, Spain; 4 Department of Nursing Science, Physiotherapy and Medicine, Hum-498 Research Team, Centre for Neuropsychological Evaluation and Rehabilitation, University of Almería, Almería, Spain; IUMPA - Universitat Politecnica de Valencia, SPAIN

## Abstract

The global economic recession is relevant in public administration, especially in terms of the human factor. If we pretend to empower people as a resource, a key aspect is the perception of equity in their relationships. Previous research has shown how a positive shared interpersonal justice climate (IJC) in a work team impacts employee well-being, affecting the level of engagement and burnout. This influence is crucial in achieving positive results in the organization and for employees. The objective was to analyze the relationship between IJC and extra-role performance (ERP) and the mediating role of two indicators of well-being (burnout and engagement) in work teams. Furthermore, the study examined the Job Demands and Resources model (JD-R) including the relationship with the work family balance (WFB) of public employees. The sample was composed of 404 technical and administrative staff in a Spanish public university. The results indicated the significant relationships between the perceptions of IJC and burnout, engagement, and the two work outcomes WFB and ERP. When burnout and engagement were introduced in the regression equations, total mediation effects were produced.

## Introduction

The economic recession in recent years has affected the management of public organizations, a situation likely to persist in the coming years [1]. The most visible consequences of this crisis are the continuity of budget cuts and legal impossibility of replenishment, as well as the reduction of employees to provide services [2]. As a result, public administrations face the challenge of meeting increasing citizen demands with less staff without reducing the quality of the services rendered [3].

One way to deal with the increasing demands of work with reduced resources is to implement practices that improve interpersonal justice climate (IJC), which affects well-being and extra-performance behaviors [4–6], while improving perceptions of work-family balance (WFB)[7–9].IJC is essential when studying group relationships at the workplace [10]. In general, this construct refers to employees' perception of the fairness of the treatment received from the organization [11, 12]. Previous studies have shown the influence of this variable on aspects related to the improvement of the work context and organizational performance [13], as well as global elements of individuals´ well-being, such as the family environment [14].

In addition, IJC is a key element in the context of the working group in terms of the perception of equity among employees in the treatment received from those holding positions of power or who distribute resources in the organization, especially supervisors[15]. Previous studies indicate that well-managed IJC, in addition to reducing stress and negative or destructive behaviors that emerge from the perception of inequity [16], can be a resource for the company and employees [17].Social exchange theory (SET) [18] provides a strong theoretical foundation to explain why employees choose to be more or less engaged in their work. This theory argues that obligations are generated through a series of interactions between parties who are in a state of reciprocal interdependence. Extant research has indicated that organizational justice would be directly associated with the quality of social exchange between individuals and their organizations [19], and in turn may lead to employee engagement [20]. Therefore, when employees have high perceptions of justice in their organization, they are also more likely to feel obliged to be fair in how they perform their roles by giving more of themselves through greater levels of engagement [21].

However, the study of IJC has important limitations. First, this variable is an individual and emerging construct with a marked social character. When employees perceive fair interpersonal relationships, they experience positive emotions, which produce an emotional contagion of the same sign in other partners [22]. Because of this transfer, the context of work teams becomes key in understanding how perception is formed in the individual [23]. Tajfel and Turner [24], based on the theory of social identity, and Blau [25], through the theory of social exchange, support this idea. But, only a small number of studies analyze the perception of justice from a multilevel perspective [26]. Thus, it is considered a future area of research, despite having become relevant in recent years [27].

A second limitation considered is that most previous research focuses on analyzing the dyadic relations between IJC on consequent or antecedent variables, show only dichotomics interactions. To deepen the understanding of IJC, global explanatory models are needed to help understand the influences of this variable in the organizational context [28] using multilevel analysis models [29].

To deal with the increasing demands of work with reduced resources, organizations need their employees to extend beyond that required of them in their jobs, and to commit themselves personally to the achievement of collective objectives [30]. This performance is referred to in several ways, such as extra-role performance (ERP) [31], and can be characterized as the act of spontaneously generating additional efforts in the duties of the job [32]. Several studies have empirically verified the importance of ERP, highlighting its influence on the individual performance employees, group performance and organizational productivity [6].

Nowadays, organizational employees are interested in combining their personal and work lives in search of well-being in life. However, differentiated roles in the workplace and at a personal level generate conflicts [33]. The balance between professional and personal aspects contributes to well-being and is related to health and good personal functioning [34]. Creating an organizational culture that supports employees in both their work and family roles is important for employee well-being [35].

Work-Family Balance (WFB) is defined as the absence or non-existence of a conflict between the roles played by a person in his work and family life [36]. Although WFB is a recently developed concept, it is addressed in some studies [37]. For example, Kossek, Colquitt, and Noe (2001) proposed the concept of a “climate for sharing concerns” (employees are encouraged to share concerns of the family role while taking part in the work role and vice versa) and found that employees who perceived this type of support (i.e., resource) also reported positive outcomes in the form of low levels of work-family conflict (defined as interference of the family domain with the work domain or vice versa) [38]. Furthermore, high perceptions of WFB have been negatively linked to employees’ psychological strain [39]. Some research provides a global conceptualization of the work-family dyad, which is associated with employees´satisfaction and consistency in their priorities [40]. As such, it is considered a global assessment of the interaction between work and family [7–9].

Burnout is conceptualized as a three-dimensional construct composed of emotional exhaustion, cynicism, and perception of professional inefficiency or labor incompetence [41].Individuals who experience burnout suffer from emotional exhaustion which depletes resources [42]. This process of deterioration also implies the appearance of cynicism. Cynical workers are less likely to exert extra effort on behalf of others, decreasing ERP [43, 44].However, the effects of burnout outweigh the work environment. Recent studies show the negative additive effect of this negative emotional state and WFB [45].

Engagement is defined as a persistent state of positive motivation of employees in the performance of their duties and is conceptualized as a three-dimensional construct characterized by high levels of energy, willingness to invest effort in work, and persistence in reaching proposed objectives [46].The link between engagement and WFB has also recently emerged in research, which indicates the mediating effect of engagement on WFB and organizational outcomes [47].The JD-R model is a suitable framework in the study of organizational context. It is supported by studies in different countries and occupational groups and has been successfully adapted to multilevel analysis studies [48, 49]. This model assumes that in any workplace associated factors affect employee burnout and engagement in two ways: a process of health deterioration and a motivational process. In the first process, job demands predict the occurrence of burnout, which is associated with negative effects for the organization and employees. The motivational process links the existence of labor resources with the emergence of employee engagement, which leads to positive results for employees and the organization [50].

**Fig 1:**
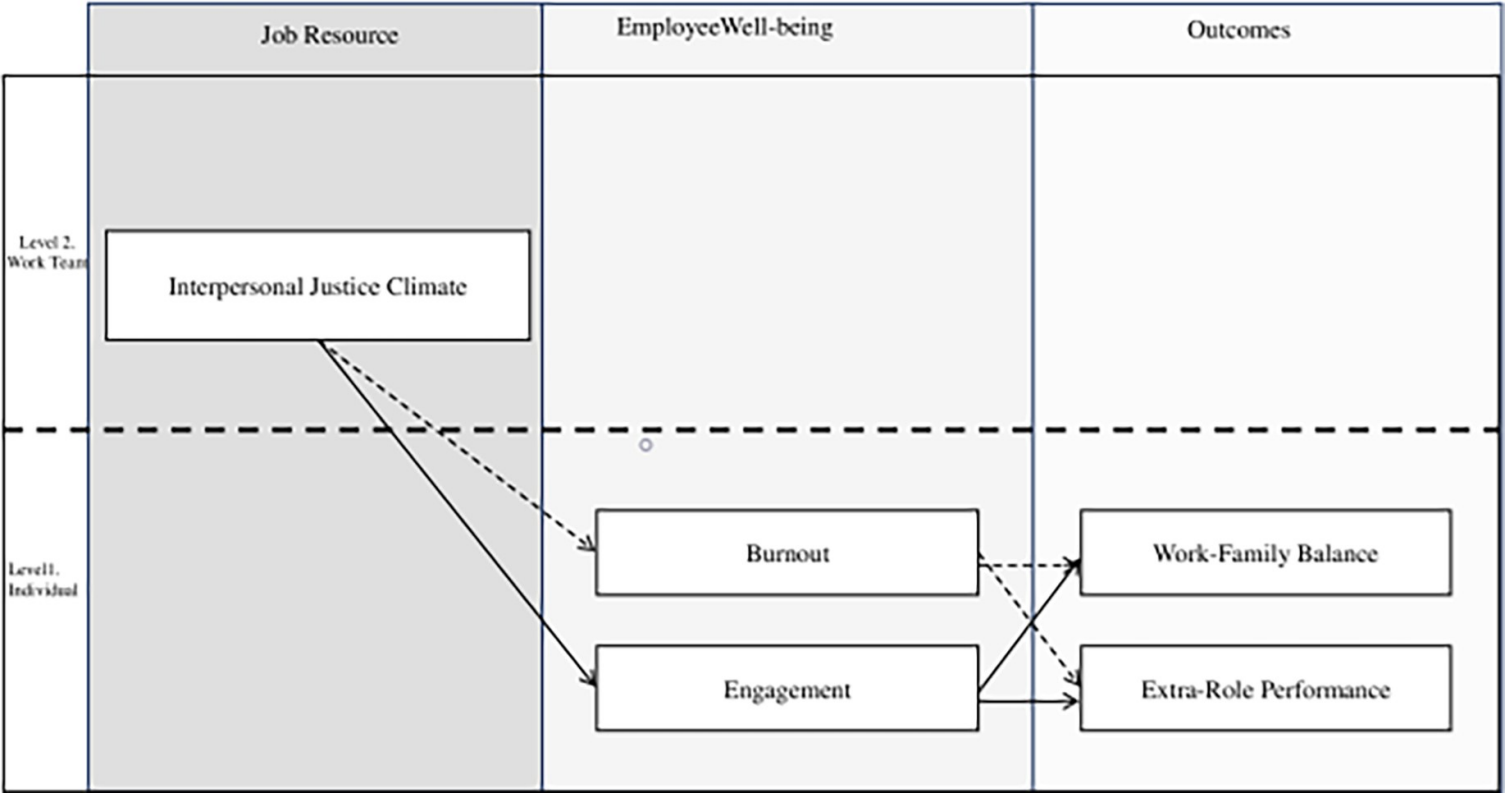
The job demands-resources model applied to the hypothesis model.

This research adapts the JD-R model to study how perceptions of IJC aggregated at the work team level influence individual ERP and WFB, considering the mediating effects of burnout and engagement as indicators of employee well-being (Fig 1).In this sense, IJC is considered an organizational job resource with the capacity to stimulate personal growth, learning, and development [51].Through the motivational process in the JD-R model, IJC affects workers’ well-being by increasing their perceptions of engagement while reducing burnout, since it can buffer the impact of strain. This motivational process will influence two positive results: increasing the worker’s extra-role performance and, at the individual level, enhancing their perception of control over the environment affecting the work-family balance.

Our research model is novel in four ways. First, we analyze the perception of justice from a multilevel perspective. Second, this study uses a global explanatory model to help understand the influence of IJC on positive outcomes, using a well-being mediation model. Third, our model extends beyond the performance usually required in a job, to personal commitment to the achievement of collective objectives, by analyzing extra-role performance and the combination of personal and work lives, in search of well-being in work life. Fourth, this research focuses on public administration, a complex organizational context that must face the challenge of meeting increasing citizen demands with less staff and without reducing the quality of services rendered [3].

Therefore, the following hypotheses were considered:

H1: IJC will have a significant and positive effect on WFB and employees' ERP and positive influence on engagement.

H2: IJC will have a significant and negative effect on burnout.

H3: Burnout will mediate the relationship between IJC and WFB and between IJC and ERP.

H4: Engagement will mediate the relationship between IJC and WFB and between IJC and ERP.

## Materials and methods

### Participants and procedure

In this descriptive, cross-sectional study, data were collected through online questionnaires. In total, 476 university employees were invited to participate, who were distributed in 33 work teams with an average unit size of 18.08 (SD = 10.86). Each participant received a questionnaire and instructions for completing it (97.90% response rate). Of all the questionnaires collected, 404 (84.87%) were correctly completed and could be included in the analysis. The Ethical Review Committee at the University of Almería (Spain) approved the study. All subjects gave written informed consent in accordance with the Declaration of Helsinki.

The final sample of this study consisted of 404 employees. Age was distributed within four intervals (from 26 to 35 years = 3%, 36 to 45 = 45%, 46 to 55 = 43%, and 56 or older = 9%). Regarding sex, 52% were men and 48% women. As for the legal relationship, 97% had the status of civil servants, and 3% employment contracts. Only 17% performed supervisory functions. The level of education was distributed in these categories: without completed 150 education, 5% primary school, 36% secondary school 29%, and higher education 30%.

### Instruments

*Interpersonal Justice Climate*was measured using the Spanish adaptation of Colquitt’s Organizational Justice Scale [52] made by Díaz-Gracia, Barbaranelli, & Moreno-Jiménez [53]. IJC is a dimension of organizational justice and reflects the degree to which people are treated with politeness, dignity, and respect by authorities or third parties involved in executing procedures or determining outcomes. Response options are delivered on a Likert scale ranging from 1 (to a small extent) to 5 (to a large extent), with higher scores indicating a higher level of perceived interpersonal justice. The psychometric characteristics of the original scales, as far as the factorial structure is concerned, were examined in two different samples, one composed of 301 university students and the other composed of 337 employees in a field setting, where a four-factor model was confirmed in both samples to be the best fit. This dimension comprises four items: i.e.,“The following items refer to (the authority figure who enacted the procedure). To what extent:Has (he/she) treated you with respect?”This scale obtained a Cronbach’s alpha reliability of .94.

*Burnout* was evaluated by means of the Spanish adaptation [54] of the Maslach Burnout Inventory (MBI) developed by Maslach and Jackson [55]. This tool has three underlying dimensions: exhaustion, which is composed of three items (i.e., “I feel emotionally drained by my work”); cynicism, which is composed of seven items (i.e., “I have become less enthusiastic about my work”); and efficacy, which consists of three items (i.e., “I can effectively solve the problems that arise in my study/work”). All items are scored on a five-point frequency rating scale ranging from 1 (“strongly disagree”) to 5 (“strongly agree”). High scores on exhaustion and cynicism and low scores on efficacy are indicative of burnout (i.e., all efficacy items are reversibly scored). The internal consistency (Cronbach’s α) of the scale was .8.

*Engagement* was measured using the Utrecht Work Engagement Scale (UWES) [56]. The items of the UWES are grouped into three subscales that reflect the underlying dimensions of engagement: vigor (six items: i.e.,“At work, I feel full of energy”), dedication (five items: i.e.,“My job inspires me”), and absorption (six items: i.e.,“I am immersed in my work”). All items are scored on a seven-point frequency rating scale ranging from 0 (never) to 7 (always).The internal consistency of the scale was .94.

*Work Family Balance* was measured using the WFB questionnaire by Carlson et al. [7]. The questionnaire consists of six items designed to represent the definition developed by Carlson (2009) of work-family balance that refers to the extent to which an individual is meeting negotiated role-related expectations in both the work and family domains [9]. Therefore, each item includes a reference to the expectations or negotiation of roles and each item taps the perspective of an external party to capture what other people expect from the focal individual (people, supervisors, family members, co-workers). A sample item is “I do a good job of meeting the role expectations of critical people in my work and family life.” All items are scored on a five-point Likert scale ranging from1 (strongly disagree) to 5 (strongly agree).The internal consistency of the scale was .93.

*Extra-role performance* was measured using the dimension in the Work Unit Performance Scale by Goodman & Svyantek [57]. The scale consists of three items analyzing actions that go beyond what is stated in formal job descriptions and increase organizational effectiveness (i.e., “I willingly attend functions not required by the organization but that help in its overall image”).Participants responded on a seven-point scale ranging from 1 (strongly disagree) to 7 (strongly agree). The internal consistency of the scale was .93.

## Statistical analysis

### Aggregation index

IJC has been analyzed as a predictor at the level of the work teams making up the university. For this, it is necessary to evaluate the degree of agreement in the perceptions of members that comprise these teams. The ICC1 and ICC2 indices were calculated for this purpose [58, 59]. From a consensus-based approach, the Average Deviation Index (ADM (J) [60] and Rwg (J) [61]) were analyzed. In addition, an analysis of variance (ANOVA) was performed to determine if there was significant discrimination between the scores displayed by the distinct groups.

The ICC1 and ICC2 indices obtained for the IJC variable were .76 and .90, respectively. The average value of the ADM (J) was .79, while the value of the Rwg (J) was .67. These results indicate the adequacy of the aggregation of the perception values of IJC among the work teams comprising the sample studied.

### Linear hierarchical multilevel model

To test the hypotheses, a linear hierarchical model was employed [62] in two levels of analysis (group and individual), including two mediator variables at the individual level (engagement and burnout, model 2-1-1). The model includes unit size as a control variable. The choice of this type of variable is of great relevance, as inclusion of such variables in most cases implies the contamination of the variables of interest or the distortion of the relationships observed between them [63]. Unit size is a determining factor that can affect the linear hierarchical model [64]; therefore, controlling its effect on the criterion variables is of great importance for the validity of the results found.

The effects of mediation can be estimated erroneously when different values are obtained inside and outside the group in terms of the magnitude. In this study we employed the analysis of hierarchical models described in Zhang [65]. This procedure allows a more accurate test of cross-effects, and reduces the problems of estimation at the aggregate level of analysis [66]. Once we obtained each coefficient of the corrected models, mediation was tested using the test´s step-by-step approach of the Sobel test [67].

## Results

The mean, standard deviation, internal consistency and correlations between variables are provided in Table 1. All correlations were significant and demonstrated the expected pattern of interrelations between the study variables. IJC at the work team level and burnout correlated negatively and significantly (r = -.44, p <0.001). IJC at the work team level was positively and significantly related to engagement, ERP, and WFB, with correlations of .41, .35 and .30, respectively. As expected, these three variables were negatively and significantly interrelated with burnout (r = -.58, -.43, -42, respectively). On the other hand, engagement demonstrated positive and significant correlations, with ERP (r = .54, p <0.001) and WFB (r = .51, p <0.001). The two outcome variables (ERP and WFB) were positively and significantly correlated (r = .45, p <0.001).

**Table 1:** The means, standard deviations, internal consistencies, and correlations between variables.

Variables	M	SD	1	2	3	4	5	6
1. IJC	3.86	.87	(.94)					
2. Burnout	2.07	.70	-.44***	(.80)				
3. Engagement	4.32	1.31	.41***	-.58***	(.94)			
4. ERP	4.84	1.04	.35***	-.43***	.54***	(.93)		
5. WFB	4.17	.79	.30***	-.42***	.51***	.45***	(.93)	

Note. Internal consistencies on the main diagonal. N level 1: 476 public employees; N level 2: 33 work units

***p < .001.

The hierarchical regression model (Table 2) demonstrated the mediating effect of burnout on the relationship between IJC, WFB, and ERP. First, the results show the positive and significant influence of IJC at the work team level on WFB (β = .63, p < .01), and ERP (β = .64, p < .05). The results also indicate the significant and negative influence of IJC at the work team level on burnout, (β = -69, p < .001).None of the three variables influenced the size variable of the work unit, which was used as a control variable (WFB: β = .00, p>.05, ERP: β = .15, p>.05, burnout: β = -.01, p >.05). The second step showed that the significant effects of IJC at the work team level on WFB and ERP disappear (WFB: β = .28, p>.05, ERP: β = -.02, p>.05).The non-significant effects of the size of the work team were maintained. These results confirm the total mediation of burnout on IJC at the work team level, WFB and ERP.

**Table 2:** The results for burnout hierarchical regression models.

	Mediator			WFB			ERP	
Step and variable	Β		*SE*	Β		*SE*	β	*SE*
1. IJC	-.69***		.14	.63**		.19	.64*	.22
Unit size	-.01		.00	.00		.00	.15	.00
2. IJC				.28		.25	-.02	.29
Unit size				.00		.00	-.00	.00
Burnout (M)				-.43***		.05	-.56***	.28

*Note*. M = Mediator. N level 1: 476 public employees; N level 2: 33 work units

* *p*< .05.

**p< .01.

***p< .001.

The second regression model provided the results of the mediating effect of engagement on the relationships between IJC on WFB and ERP. The results provided in Table 3, indicate the positive and significant influence of IJC on WFB (β = .63, p < .01) and ERP (β = .64, p < .05). Furthermore, the results show the significant and positive influence of IJC on engagement(β = .27, p < .001). None of the three variables influenced the size of the work team (WFB: β = .00, p. ns; ERP: β = .15, p>.05, burnout: β = .01, p>.05). The second step demonstrated that the effects of IJC at the work team level on WFB and ERP were no longer significant (WFB: β = .14, p>.05, ERP: β = -0.4, p>.05). In this step, the non-significant effects of the size of the work team were repeated. These results confirm the total mediation of engagement in the influence of IJC at the work team level on WFB and ERP.

**Table 3:** Results for engagement hierarchical regression models.

	Mediator			WFB			ERP	
Step and variable	Β		*SE*	Β		*SE*	Β	*SE*
1. IJC	.27***		.28	.63**		.19	.64*	.22
Unit size	.01		.00	.00		.00	.15	.00
2. IJC				.14		.20	-.04	.27
Unit size				.00		.00	.00	.00
Engagement (M)				.28***		.02	.40***	.03

*Note*. M = Mediator. N level 1: 476 public employees; N level 2: 33 work units

* *p*< .05.

**p< .01.

***p< .001.

To formally evaluate the indirect specific effects, the bootstrapping technique has been used, following the procedure proposed by Preacher and Hayes (2004) [68]. The results of the confidence intervals for indirect effects of interpersonal justice are summarized in Table 4 for WFB and ERP.

**Table 4:** Confidence intervals for the indirect effects of the interpersonal justice scheme in the WFB and ERP, using the bootstrapping procedure.

	Coef.	*SE*	Bootstrap, 95% IC	
			Lower	Higher
WFB				
1. Engagement	.28	.02	.22	.33
2. Burnout	-.43	.05	-.53	-.32
ERP				
1. Engagement	.40	.03	.33	.48
2. Burnout	-.56	.07	-,71	-,41

As can be seen, all indirect effects for interpersonal justice of engagement and burnout are significant.

## Discussion

This study confirmed the relationship between IJC, and ERP and the mediating role of indicators of well-being (burnout and engagement). On the other hand, the JD-R model included the relationship with the WFB of public employees in this study. For this purpose, our research model, an expanded JD-R model [48, 56], was employed to validate the hypotheses of this study.

Our first hypothesis argued that IJC would have a significant and positive effect on WFB and ERP. The results confirmed this association (β = .63, p < .05), suggesting that improved IJC will produce positive results for WFB, and the increase in perceptions of IJC will increase employees´ ERP (β = .64, p < .05).

The second hypothesis proposed the significant and negative influence of IJC on burnout. Ours results demonstrated that improving the IJC of work team members reduces their level of burnout (β = -69, p < .001).

Other hypotheses posited the mediating effects of burnout on the relation between IJC in work teams, WFB, and ERP respectively. Thus, when burnout is included in the regression equations, the influence of IJC on WFB in work teams is no longer significant (β = .28, n.s.). The same is true when burnout is included in the equation as a mediator between IJC and ERP (β = -.02, n.s.). This suggests that IJC influences WFB and ERP, but only through the impact on burnout. On the other hand, the results indicate that IJC has a significant and positive effect on engagement (β = 1.27, p < .001).

The last hypothesis postulated the mediating effects of engagement on the influence of IJC on WFB and ERP, respectively. The results indicated that when engagement is included in the regression equation, the influence of IJC on WFB and ERP are not significant (β = .14, n.s. and β = -04, n.s., respectively).

### Theoretical implications

One contribution of the current studyis the reinforcement of the multilevel perspective in organizational justice research. Few studies have analyzed this variable from a multilevel model [26, 67], especially in the public administration context. Along these lines, Mayer [69] found that individual perceptions of justice had greater influence on outcome variables when the perception was shared with other members of the work team units; however, he did not explain the reasons for this greater influence. The JDR model allows us to analyze the influence of shared perceptions as aspects that vary the burnout-engagement dichotomy. This provides us variation in subjects´ perception of well-being, which explains the greater effort required when developing performance-oriented behaviors.

Another relevant contribution of the current research is the confirmation of the global explanatory model in the IJC study beyond organizational context [28]. Our result showsthat the effects of the IJC and well-being dichotomy (burnout-engagement) go beyond the job environment and the efforts required in a work role. To date, most research has focused on the effects of perceived justice on aspects such as satisfaction, performance, help behaviors, propensity to leave the job, or customer satisfaction [70,71]. However, the results of this research show how an organizational context variable (IJC) can affect the lives of employees beyond their professional lives (WFB) and improve their willingness to strive for achievements that surpass job requirements (ERP).

### Practical implication

The results of this study have important practical implications for the management of administrative and service staff in a public administration. First, the way in which human resources are managed is crucial to achieving employees´ well-being. The results demonstrate the importance of IJC in developing "engaged" employees [67]. This will increase extra-role behavior, as they are more willing to expend extra effort in their jobs [72]. When employees perceive that they are treated unfairly, their experience were tension or stress.

Second, the results lead us beyond the professional field. Being able to balance personal and family life is a key element in achieving well-being. The results presented in this paper show that employees who perceive a fair working context in interpersonal relationsincreasetheir extra-role effortsdue to a positive perception of balance between the professional and personal aspects of their lives. As previous studies have found, this relation influencesemployees’quality of life, stress, depression, and departure intentions [73].

### Limitations and future research

However, the results obtained in this study should be considered under the following limitations. First, the results were obtained from self-reports and could be affected by common method variance;yet Harman test results [74] showed that exploratory factor analysis with all study variables produced values in the first factor that did not exceed 50% of the variance between the variables (41.15%) [75]. In addition, a poor fit of the model was revealed (*X*^*2*^ = 10561,792, p < .001), which means that common method variancewould not be a serious deficiency in this study. Also, the use of intersubjective responses in the work teams (aggregate responses) could mitigate this effect. Second, the sample is very specific, limited to the collective of administration and services staff at a public university. Therefore, the results mustbe generalized to other types of organizations with caution. However, the results are interesting as inputs for interventions to improve employees´ well-being and develop healthy public organizations. Third, the design of the study has a transverse nature, which prevents us from drawing conclusions about the temporal order of effects and causal relationships. However, the longitudinal effects of the test were not the main objective of this study, since we tested a model of the multilevel mediation of employee well-being.

Following the above limitations, we suggest other forms of data collection using records obtained through direct observation or evaluation interviews of critical incidents. This would provide complementary measures to corroborate the goodness of the data used. Second, it could be convenient to increase the sample spectrum of the study (e.g. compare samples from public and private administration), for a multivariate investigation. It is necessary to carry out longitudinal studies that enable an analysis of the evolution of and causal influences in improving justice about well-being, group performance, and WFB.

Finally, other variables could be incorporated in future studies to create extended models. To this end, leadership could be a critical variable. It is well known that the behavior of leaders has an important influence on employees. Accordingly, different leadership styles (e.g., transformational) could be investigated as precursors or obstacles of ICJ, ERP, and WFB. Another relevant variable involves the deep values of the organization,with respect to culture. For example, Erdogan, Leden,and Kraimer (2016) pointed out the mediation effect of culture between leader-member exchange and organizational justice [76]. Therefore, cultural values can add valuable information for understanding the impact of IJC in the organizational environments of public employees[77].

## Conclusions

Despite its limitations, the current research study contributes to the literature by examining the role of the shared perception of interpersonal justice and well-being of public employees in understanding how to improve ERC and WFB. Public employees display voluntary behaviors to help the organization and their colleagues and reduce the perception of conflict between work and personal life,because employees perceive fairness in the treatment received from the organization.

## Supporting information

S1 Dataset Renamed_bde93. (SAV)
